# Impact of electrocardiogram monitoring on the frequency of tracheal intubation at birth^✰^

**DOI:** 10.1016/j.jped.2026.101504

**Published:** 2026-02-08

**Authors:** Thalles de Souza Freire, Mandira Daripa Kawakami, Maria Fernanda de Almeida, Ruth Guinsburg

**Affiliations:** Universidade Federal de São Paulo, Escola Paulista de Medicina, São Paulo, SP, Brazil

**Keywords:** Electrocardiography, Heart rate, Infant, newborn, Infant, premature, Intermittent positive-pressure ventilation, Resuscitation

## Abstract

**Objective:**

To determine whether the use of an electrocardiogram monitor in newborns receiving positive pressure ventilation (PPV) at birth affects the frequency of tracheal intubation in the delivery room.

**Methods:**

Retrospective cohort of liveborn infants without congenital anomalies, with gestational age (GA) ≥ 23 weeks and birth weight ≥ 400 *g* who received PPV at birth, from 2014 to 2022. Newborns were stratified by GA (< 34 or ≥ 34 weeks) and by use or non-use of an electrocardiogram monitor during resuscitation. Logistic regression was used to assess the association between electrocardiogram monitoring and outcomes of interest for each GA group.

**Results:**

Among 5622 live births, 516 met the inclusion criteria; 224 (43 %) were monitored, and 292 (57 %) were not. The frequency of tracheal intubation was similar between monitored and non-monitored groups: ≥ 34 weeks - 13 % vs. 14 %; < 34 weeks - 43 % vs. 43 %. Electrocardiogram monitoring increased the odds of initiating PPV with a face mask ≥ 60 seconds after birth by 2.45-fold (95 % CI: 1.08–5.54) for GA ≥ 34 weeks and by 2.72-fold (95 % CI: 1.13–6.59) for GA < 34 weeks, after adjustment for umbilical cord clamping time, year of birth, and birth weight.

**Conclusion:**

Electrocardiogram monitor use did not reduce the frequency of tracheal intubation and was associated with delayed initiation of PPV with a face mask.

## Introduction

Positive pressure ventilation (PPV) is the most important and effective procedure in neonatal resuscitation. A study conducted in Tanzania showed that, among 7862 live births, 14 % required some assistance after birth, and 6.8 % received PPV [1]. In Brazil, data from 20 public university hospitals participating in the Brazilian Network on Neonatal Research between 2014 and 2020 showed that, among 3644 extremely preterm infants, 83 % received PPV in the delivery room, and among 4870 very preterm infants, 59 % were ventilated [2]. PPV should be initiated within the first 60 s after birth, so-called “Golden Minute”, with an increase in heart rate (HR) being the main indicator of procedural effectiveness. Therefore, fast and accurate methods for monitoring HR at birth are essential for successful neonatal resuscitation [3,4].

HR assessment in the first minutes of life can be performed by palpating the umbilical cord, auscultating the precordium with a stethoscope, detecting the pulse by pulse oximetry, or measuring the heart’s electrical activity using an electrocardiogram monitor (ECG) [3, 4, 5, 6]. A study involving 26 neonates with a mean gestational age (GA) of 38 weeks showed that HR was underestimated in 100 % of evaluations performed by palpation and auscultation compared with ECG [7]. Pulse oximetry provides simultaneous and continuous data on oxygen saturation and HR, but it may delay HR detection by 122 s or more (vs. 24 s with the ECG), leading to delayed initiation of resuscitation procedures or continuation of unnecessary interventions [8, 9, 10, 11]. The ECG is considered the “gold standard” because it is accurate and reliable, although it does not provide information on oxygenation [12, 13, 14, 15]. Thus, research shows that while traditional clinical methods tend to underestimate HR, the ECG is more accurate and recommended to ensure appropriate interventions during neonatal resuscitation [16,17].

Continuous HR monitoring facilitates decision-making during resuscitation, contributing to better clinical outcomes, such as reduced time to achieve HR ≥100 bpm and decreased in-hospital mortality [18]. In Brazil, the Neonatal Resuscitation Program of the Brazilian Society of Pediatrics (Brazilian NRP) has recommended its use since 2016 for newborns with HR <100 bpm or without regular respiratory movements after the initial steps of resuscitation. While one professional initiates PPV, another applies the pulse oximeter sensor and the three electrodes of the ECG. In 2022, these guidelines were updated to recommend that for newborns < 34 weeks’ gestation, ECG use should always be implemented, regardless of the need for PPV [4,5].

Despite these recommendations, there is still a lack of clear evidence on how the speed and accuracy of measurements provided by ECGs impact clinical outcomes, such as the timing of neonatal resuscitation procedures, admission to the neonatal intensive care unit (NICU), and neonatal survival [18, 19, 20]. In this context, the present study aimed to assess, in newborns without congenital malformations who received PPV in the delivery room, whether the use of ECG with continuous HR display, compared with HR assessment by auscultation/palpation and/or pulse oximetry, altered the frequency of tracheal intubation.

## Methods

This was a retrospective cohort study conducted at a tertiary-level perinatal center in a public university hospital. The investigation included all liveborn infants with GA ≥ 23 weeks and birth weight (BW) ≥ 400 g who received PPV via face mask in the delivery room between January 1, 2014, and December 31, 2022. The study was approved by the Committee of Ethics in Research of Universidade Federal de São Paulo (CAAE 66,431,322.5.0000.5505).

Newborns were excluded if they had congenital malformations, were born outside the obstetric unit, were initially ventilated with a tracheal tube without prior PPV via face mask, or started PPV after the fifth minute of life due to irregular breathing or respiratory distress. Included infants were classified into two GA categories: < 34 weeks and ≥ 34 weeks. They were further subdivided according to the method of HR assessment at birth: “Use of ECG” and “Non-use of ECG.” The first group included infants whose HR was monitored in the neonatal resuscitation room with ECG and pulse oximetry, in addition to clinical assessment by precordial auscultation with a stethoscope. The second group included those whose HR was monitored by pulse oximetry and/or precordial auscultation. The indications for HR monitoring using ECG followed the Brazilian NRP guidelines. Accordingly, before 2016, ECG monitoring was rarely used in the delivery room. From 2016 to May 2022, ECG-based HR monitoring was recommended for newborns requiring PPV at birth, regardless of gestational age. From July to December 2022, ECG monitoring was indicated for neonates ≥ 34 weeks’ gestation who received PPV at birth and for all neonates < 34 weeks’ gestation as part of the initial steps of postnatal care [4,5].

Data collection was performed using the “Neonatal Health Information System” of the institution, which has prospectively recorded demographic and clinical information of liveborn infants on a daily basis since 2003. Missing data in the system were complemented by reviewing physical medical records. Collected information included maternal demographic, clinical, and obstetric characteristics. For newborns, GA, sex, anthropometric data, and details of stabilization and resuscitation interventions at birth, as well as Apgar scores at 1 and 5 min, were recorded. Clinical outcomes included air leak syndrome within the first 48 h, admission to the neonatal intensive care unit (NICU) for infants ≥ 34 weeks, and discharge status (alive or deceased).

Sample size was calculated based on the study by Shah et al. [19], considering a 12 % reduction in the need for tracheal intubation with ECG use, statistical power of 80 %, and alpha error of 5 %, resulting in a required sample of 260 newborns in both ECG and non-ECG groups. Although this was a retrospective cohort study using existing data, a sample size calculation was performed to ensure adequate statistical power to detect a clinically meaningful difference in the primary outcome. This calculation was conducted during the design phase to confirm that the available cohort was sufficient to address the study hypothesis and to minimize the risk of a type II error.

Continuous variables were presented as mean and standard deviation or median and interquartile range, and compared using Student’s *t*-test or Mann–Whitney U test. Categorical variables were presented as frequencies and compared using the chi-square (χ²) or Fisher’s exact test. Multivariate logistic regression models were used to evaluate the association between ECG use and outcomes for each GA group (≥ 34 and < 34 weeks). Independent variables with a significance level of *p* < 0.10, along with the variable of interest (ECG use), were included in the models. Given that the study spanned nine years and encompassed changes in Brazilian Neonatal Resuscitation Program (NRP) guidelines, all models were additionally adjusted for study period (before 2016 and 2016–2022). Results were expressed as adjusted odds ratios with 95 % confidence intervals. Statistical analysis was performed using The Jamovi Project (Version 2.6, 2025).

## Results

**Figure 1:**
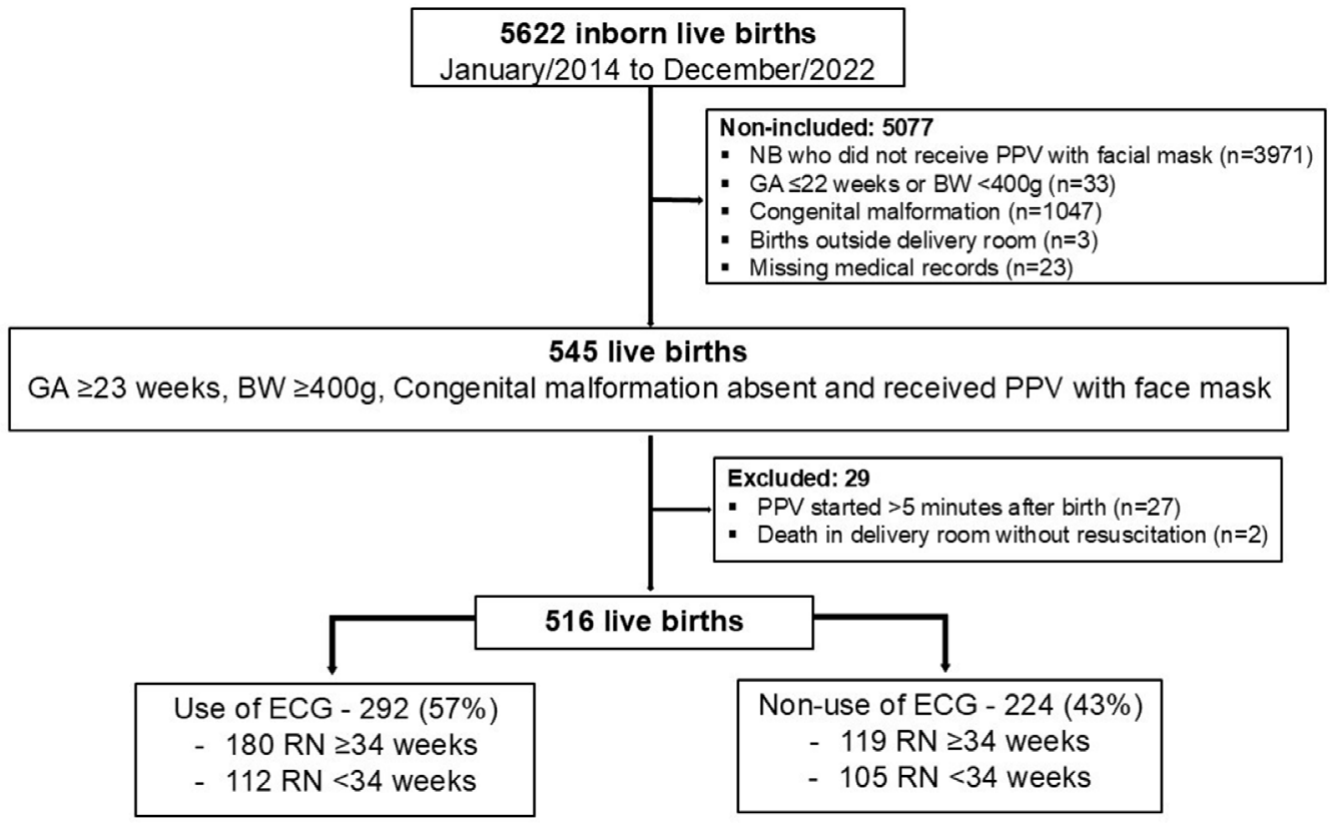
Flow diagram of the included infants. BW, Birth weight; ECG, Electrocardiogram monitor; GA, Gestational age; PPV, Positive pressure ventilation.

Among the 5622 live births from January 2014 to December 2022, 5077 did not meet the inclusion criteria. Of the 545 potentially eligible liveborn infants, 27 were excluded because PPV was initiated after 5 min of life, and 2 were excluded because they died in the delivery room without receiving advanced resuscitation procedures. Thus, 516 liveborn infants were analyzed: 292 (57 %) in the “Non-use of ECG” group and 224 (43 %) in the “Use of ECG” group. Of the 516 infants analyzed, 299 (58 %) had GA ≥ 34 weeks, and 217 (42 %) had GA < 34 weeks (Figure 1).

**Figure 2:**
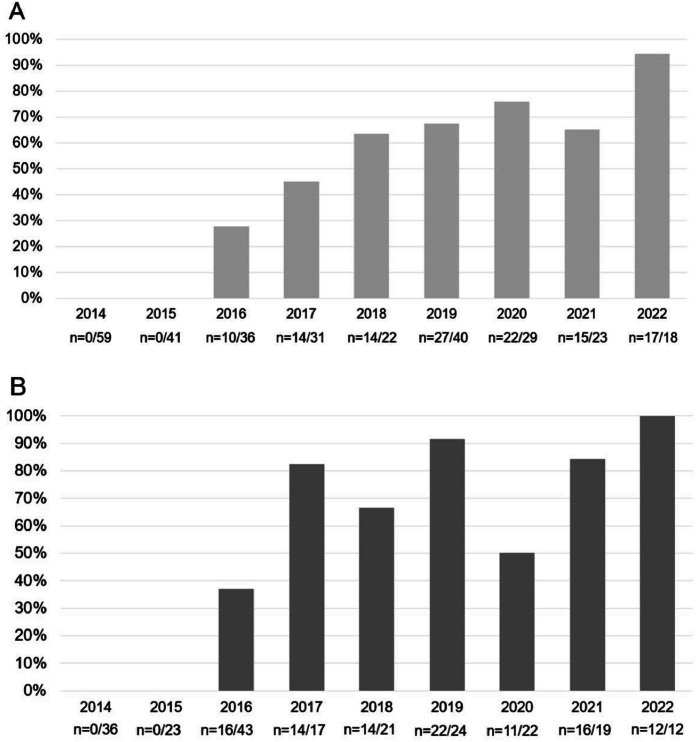
Frequency of ECG use in newborns who received PPV by face mask in the delivery room, according to year of birth. A – 299 neonates ≥ 34 weeks’ gestation; B – 217 neonates < 34 weeks’ gestation.

Figure 2 shows the progressive increase in ECG use over the years, reaching 94 % among newborns ≥ 34 weeks’ gestation and 100 % among those < 34 weeks who received PPV via face mask in 2022 (χ² for trend; *p* < 0.001 for both groups).

The main characteristics of the 299 liveborn infants ≥ 34 weeks’ gestation who received PPV via face mask according to ECG use [*n* = 119 (40 %)] or no ECG use [*n* = 180 (60 %)] are presented in Table 1. At birth, the mean GA was 38 weeks, and BW was 2900 g in both groups. In infants monitored with ECG, PPV via face mask was initiated at a mean of 45 ± 22 s after birth, whereas in those without ECG monitoring, PPV began earlier, at 34 ± 13 s (*t*-test; *p* < 0.001). In addition, 31 % of ECG-monitored infants initiated PPV after the first minute of life, compared with 10 % of those without ECG monitoring (χ²; *p* < 0.001). Regarding the primary outcome, tracheal intubation was performed in 13 % of ECG-monitored and 14 % of non-ECG infants, with no significant difference between groups.

**Table 1:** Characteristics, delivery room interventions, and outcomes of newborns according to gestational age and electrocardiogram monitor use.

	Gestational age ≥ 34 weeks		p-value	Gestational age < 34 weeks		p-value
	Use of ECG			Use of ECG		
	NO (*n* = 180)	YES (*n* = 119)		NO (*n* = 112)	YES (*n* = 105)	
Maternal and neonatal characteristics						
Maternal age (years)*	30 ± 7.5	30 ± 6.9	0.724^b^	30 ± 5.9	30 ± 7.1	0.658^b^
Multiple gestation	22 (12 %)	8 (7 %)	0.121^a^	42 (38 %)	23 (22 %)	**0.012**^a^
General anesthesia	16 (11 %)	16 (17 %)	0.160^a^	16 (18 %)	11 (13 %)	0.441^a^
Cesarean delivery	147 (82 %)	91 (76 %)	0.275^a^	91 (81 %)	85 (81 %)	0.955^a^
Gestational age (days)⁎⁎	266 (254–277)	266 (255–276)	0.670^c^	201 (184–218)	207 (188–220)	0.382^c^
Male sex	96 (53 %)	53 (45 %)	0.137^a^	46 (41 %)	58 (55 %)	**0.037**^a^
Birth weight (grams)*	2931 ± 700	2864 ± 653	0.684^b^	1109 ± 527	1183 ± 579	0.434^b^
Year of birth 2014–2016	126 (70 %)	10 (8 %)	**<0.001**^a^	86 (77 %)	16 (15 %)	**<0.001**
Interventions in DR and outcomes						
Immediate cord clamping	148 (90 %)	111 (94 %)	0.247^a^	93 (91 %)	98 (94 %)	0.406^a^
Indication of PPV						
*HR <100bpm at birth*	109 (61 %)	64 (54 %)	0.246^a^	77 (69 %)	80 (76 %)	0.221^a^
*Apnea or irregular respiration*	170 (94 %)	113 (95 %)	0.847^a^	104 (93 %)	99 (94 %)	0.228^a^
PPV equipment			0.708^a^			0.893^a^
*Self-inflating bag*	153 (85 %)	103 (87 %)		8 (7 %)	8 (8 %)	
*T-piece*	27 (15 %)	16 (13 %)		104 (93 %)	97 (92 %)	
Initiation of PPV with face mask (*sec*.)*	34 ± 13	45 ± 22	<0.001^b^	36 ± 18	47 ± 24	**<0.001**^b^
Initiation of PPV with face mask ≥60 *sec*.	17 (10 %)	35 (31 %)	<0.001^a^	15 (14 %)	38 (37 %)	**<0.001**^a^
Duration of PPV with face mask (*sec*.)⁎⁎	30 (30–60)	60 (30–90)	0.006^c^	60 (30–60)	60 (30–90)	0.193^c^
Tracheal intubation	25 (14 %)	15 (13 %)	0.750^a^	48 (43 %)	45 (43 %)	1.000^a^
Initiation of PPV with tracheal tube (*sec*.)⁎⁎	150 (90–300)	180 (120–194)	0.217^c^	120 (90–210)	180 (120–300)	**0.025**^c^
Chest compressions and/or Epinephrine	3 (2 %)	1 (1 %)	0.543^a^	8 (7 %)	8 (8 %)	0.893^a^
Apgar score						
1st minute*	6 ± 2	5 ± 2	**0.032**^b^	5 ± 2	5 ± 2	0.140^b^
5th minute*	8 ± 1	8 ± 1	0.110^b^	8 ± 2	7 ± 2	0.829^b^
Admission to NICU	116 (64 %)	76 (64 %)	0.919^d^	110 (98 %)	104 (99 %)	0.599^a^
Air leak in the first 48 h after birth	1 (1 %)	1 (1 %)	1.000^d^	8 (7 %)	3 (3 %)	0.150^a^
Hospital death	3 (2 %)	2 (2 %)	0.993 d	33 (29 %)	25 (24 %)	0.347^a^

⁎ mean ± standard deviation.

⁎⁎ median (interquartile interval).

a Chi-square.

b *t*-test.

c *Mann-Whitney*.

d *Fisher exact test;* DR, Delivery room; ECG, Electrocardiogram monitor; HR, Heart rate; NICU, Neonatal intensive care unit; PPV, Positive Pressure ventilation.

The main characteristics of the 217 liveborn infants < 34 weeks who received PPV via face mask according to ECG use [*n* = 105 (48 %)] or no ECG use [*n* = 112 (52 %)] are also shown in Table 1. At birth, the mean GA was 29 weeks and the mean BW was approximately 1100 g in both groups. In those monitored with ECG, PPV via face mask was initiated at a mean of 47 ± 24 s after birth, whereas in those without ECG monitoring, PPV began earlier, at 36 ± 18 s (*t*-test; *p* < 0.001). Furthermore, 37 % of ECG-monitored infants initiated PPV after the first minute of life, compared with 14 % of those without ECG monitoring (χ²; *p* < 0.001). For the primary outcome, tracheal intubation was performed in 43 % of newborns in both groups in the delivery room.

In the multivariate analysis for the dependent variable “tracheal intubation,” separate logistic regression models were adjusted for newborns ≥ 34 weeks and < 34 weeks’ gestation, considering as explanatory variables: GA in days, birth year (2014–2016), cesarean delivery, general anesthesia for delivery, classification as small for gestational age [21], and the variable of interest (ECG use). In both GA groups, general anesthesia for delivery was a significant risk factor for tracheal intubation, whereas ECG use was not significantly associated with the outcome (Table 2).

**Table 2:** Multivariate regression analysis, according to gestational age group.

A. Dependent variable: Tracheal intubation		
	Adjusted Odds Ratio (95 % CI)	p-value
**Infants ≥ 34 weeks’ gestation**		
Gestational age (days)	1.00 (0.97–1.02)	0.953
Year of birth 2014 to 2016	1.37 (0.56–3.35)	0.490
Cesarean delivery	0.76 (0.28–2.05)	0.588
General anesthesia	3.25 (1.32–8.03)	**0.011**
Small for gestational age	0.82 (0.17–3.88)	0.800
Use of ECG	1.01 (0.41–2.50)	0.987
**Infants < 34 weeks’ gestation**		
Gestational age (days)	0.96 (0.95–0.98)	**<0.001**
Year of birth 2014 to 2016	0.45 (0.19–1.08)	0.073
Cesarean delivery	0.53 (0.13–2.17)	0.373
General anesthesia	7.13 (2.67–19.03)	**<0.001**
Small for gestational age	1.55 (0.75–3.21)	0.238
Use of ECG	0.99 (0.43–2.30)	0.984

B. Dependent variable: Initiation of PPV by face mask ≥ 60 s after birth		
	Adjusted Odds Ratio (95 % CI)	p-value
**Infants ≥ 34 weeks’ gestation**		
Umbilical cord clamping > 60 s	10.14 (3.18–32.37)	**<0.001**
Year of birth 2014 to 2016	0.21 (0.07–0.59)	**0.003**
Birth weight < 2500 *g*	1.66 (0.79–3.49)	0.182
Use of ECG	2.45 (1.08–5.54)	**0.032**
**Infants < 34 weeks’ gestation**		
Umbilical cord clamping >30 s	5.23 (1.59–17.17)	**0.006**
Year of birth 2014 to 2016	0.44 (0.18–1.10)	0.078
Birth weight < 1000 *g*	1.47 (0.74–2.92)	0.275
Use of ECG	2.72 (1.13–6.59)	**0.026**

CI, Confidence interval; ECG, Electrocardiogram monitoring.

Given the longer time to PPV initiation with ECG use observed in the descriptive analysis, logistic regression models were also adjusted for the dependent variable “initiation of PPV via face mask ≥ 60 s after birth.” For newborns with GA ≥34 weeks, the model included birth year (2014–2016), umbilical cord clamping time > 60 s, ECG use, and BW < 2500 g. For those < 34 weeks’ gestation, the model included birth year (2014–2016), umbilical cord clamping time > 30 s, ECG use, and BW < 1000 g. In both GA groups, ECG use was associated with an increased likelihood of PPV initiation ≥ 60 s after birth (Table 2).

## Discussion

In the present study, a retrospective cohort of live births at a tertiary-level Brazilian university hospital, the use of ECG increased progressively over the nine years analyzed, with a marked rise following its recommendation by the Brazilian NRP in 2016 [22,23]. ECG use did not modify the frequency of PPV via tracheal tube and did not alter the timing of its initiation in either GA group. Contrary to expectations, initiation of PPV via face mask occurred later, with a higher frequency of initiation after the first minute of life among newborns in whom ECG was used, in both GA ≥ 34 weeks and < 34 weeks.

The study hypothesis was that ECG, as the recommended equipment for rapid and accurate HR assessment in the delivery room compared to alternative methods, would reduce the need for tracheal intubation when implemented immediately after birth in newborns receiving PPV. However, the frequency of tracheal intubation was similar among infants ventilated who did or did not use ECG, in both GA categories. The 13 % frequency of tracheal intubation in newborns ≥ 34 weeks receiving PPV was consistent with the literature [24]. In contrast, the 43 % frequency of tracheal intubation in those < 34 weeks’ gestation receiving PPV was lower than the 74 % reported for neonates < 1500 g receiving PPV in the delivery room in Brazilian Network on Neonatal Research hospitals [2]. This comparison is limited, however, as the GA range in the present study includes many infants without very low birth weight, who have a lower risk of requiring resuscitation procedures at birth. Importantly, even after adjustment for potential confounders, no association was found between ECG use and tracheal intubation in either GA group.

Similar findings regarding the limited impact of ECG on tracheal intubation rates at birth have been reported in recent international publications. A randomized controlled trial involving 40 newborns at 28–29 weeks GA showed that, although ECG displayed HR more rapidly, the intubation rate remained the same (30 %) in both groups, with and without visible ECG [25]. Another randomized trial involving 51 preterm infants at 23–30 weeks’ gestation found that ECG use in the delivery room did not reduce the frequency of neonatal resuscitation procedures [26]. In a cohort of all newborns requiring PPV or other respiratory support at birth in 2015 (pre-ECG implementation), 2017 (implementation phase), and 2021 (four years post-implementation), comparison of 2015 and 2021 showed no difference in tracheal intubation rates. According to the authors, observed variability across periods could be attributed to human factors, with minimal impact of ECG on intubation frequency [20].

Regarding other outcomes, ECG use increased by 2.45-fold (95 % CI 1.08–5.54) and 2.72-fold (95 % CI 1.13–6.59) the odds of initiating PPV via face mask ≥ 60 s after birth for newborns ≥ 34 and < 34 weeks’ gestation, respectively, after adjusting logistic models for cord clamping time, birth year, and BW. In the literature, findings regarding ECG use and potential delays in PPV initiation are variable for both term and preterm newborns. A U.S. study of 51 preterm infants < 31 weeks’ gestation showed that PPV initiation delay > 30 s occurred in 4 % of infants with the ECG screen visible and in 12 % of those with the screen hidden, without statistical significance [26]. Another U.S. study of 40 infants at 28–29 weeks’ gestation found that in the ECG group, PPV via face mask was initiated at a mean of 77 ± 16 s after birth, compared with 106 ± 144 s in the non-ECG group, also without statistical significance [25]. A population-based study in Norway, including 4876 newborns ≥ 28 weeks’ gestation, reported PPV initiation at 72 s after birth [27]. According to the authors, among infants receiving PPV, the main indication was irregular breathing, justifying the delay in PPV initiation [27]. This differs from the present study, where the main indications for ventilation were bradycardia and/or apnea and/or irregular breathing immediately after birth, with HR assessment performed by auscultation soon after initial resuscitation steps. Therefore, there is no clear justification for the association between delayed PPV initiation and ECG use.

All these studies, including the present findings, highlight the challenges in implementing international and national guidelines in actual neonatal resuscitation practice [3, 4, 5, 6,28]. Evidence indicates that initiating PPV via face mask within the first 60 s after birth is essential to prevent progression to circulatory collapse in apneic newborns [29,30]. Early intervention can stabilize HR and blood pressure, preventing brain injury and death [27]. Every 30-second delay in PPV initiation increases the risk of death by 16 %, regardless of GA, BW, or delivery mode [24].

The explanation for the observed delay in PPV initiation among ECG-monitored newborns in both GA groups is unclear. Technically, at the study institution, PPV and electrode placement for ECG are performed independently by two to three healthcare professionals, as recommended by Brazilian NRP guidelines [4,25]. Thus, electrode placement when PPV is indicated should not cause delay, as these are performed by different team members. One possible explanation for these findings is improved recording of time-related events in neonatal resuscitation precisely during the period when the ECG was implemented in the resuscitation room. From 2017, in situ simulation training in the obstetric operating room was implemented for all neonatal fellows and the obstetrics team, with post-event debriefings and monthly review of quality indicators. Therefore, improvements in documentation of events and procedures in the resuscitation room may have occurred.

Thus, the focus of improvement efforts based on these results should perhaps be directed not at the speed and skill of electrode placement but at training to ensure timely initiation of PPV within the first 60 s of life in newborns experiencing difficulties in cardiopulmonary transition at birth.

In summary, this nine-year historical cohort of neonatal resuscitation procedures found no impact of ECG use on tracheal intubation rates in the delivery room and suggests that its use may delay initiation of PPV via face mask in both term and preterm newborns, warranting further research to strengthen the evidence on the clinical outcomes of ECG use at birth.

## Funding

No funding was secured for this study.

## Conflicts of interest

The authors declare no conflicts of interest.

## Data Availability

The data that support the findings of this study are available from the corresponding author.
